# Editorial Notes

**Published:** 1920-03

**Authors:** 


### Editorial Notes.

**Proposed Amalgamation of Bristol Hospitals.**

For some considerable time a scheme has been under consideration to amalgamate or co-ordinate the chief voluntary hospitals and other medical charities of the City of Bristol, and by May 31st these various institutions will be invited to pass such resolutions as may be necessary to the attainment of this end. The decision on this great question involves great issues, and if it proves a success the event will be historic in British hospital work.

We understand that the following Institutions are to be invited to co-operate in the amalgamation: The Bristol Royal Infirmary, Bristol General Hospital, Royal Hospital for Sick Children and Women, Bristol Eye Hospital, The Convalescent Home, The Bristol Dispensary, Cossham Hospital, The Orthopædic Hospital, and the Cripples' Home and the Eye Dispensary. Those of the above electing to come into the scheme will be represented on a General Council, which shall be the central authority, presumably to co-ordinate the financial and general management of the Institutions.

The situation which has arisen in the financial state of the British Voluntary Hospitals, and which has to be reckoned with, is one of the misfortunes of the great war, for although the ever-growing needs of these hospitals created difficulties before the war, philanthropy devised means of overcoming those difficulties. And now that these difficulties have been magnified a hundredfold owing to the enormously increased numbers of those for whom treatment in hospitals has become essential and the diminished ratio of those who have hitherto supported the hospitals, many are tempted to lose heart, and join in the cry that the voluntary hospitals are doomed, although the general excellence which characterises the work they do has inspired such widespread confidence that they have become an essential part of our national life, and by the force of example have compelled a raising in the standard of all collateral State relief. Hence the stand taken by the President of the Bristol General Hospital, Mr. George A. Wills, is encouraging and timely, for he urges that the city memorial to those who have fallen in defence of the country should be the raising of a sum which shall place the voluntary hospitals on a sound financial footing. But when we consider that the preservation of the voluntary hospitals is not only a great financial problem, but involves also the equally important question of developing the teaching and training of medical practitioners and the evolution of British medicine and surgery in the soundest, most scientific manner, the responsibility of those who manage these institutions cannot end with the collection of even the enormous amount of money which will serve to remove outstanding debt and maintain them for the immediate future. Great commercial concerns demonstrate the imperative need for amalgamations and co-ordinations to save overlapping, administrative expense and impoverishing competition. But hospitals as we know them are not commercial concerns, and just as the most distinguished scientists are often the most unbusinesslike, so with hospitals, once the commercial instinct becomes the dominant factor in their work its value and attraction will dwindle, they might as well be State institutions. Yet this, to our mind, is the strongest argument for the effort now being made by those who seek to save the voluntary hospitals by bringing to bear on their business aspects the experience and organising skill of those who think imperially. The difficulty is to devise a method of re-organising the voluntary hospital system without sacrificing the initiative and the enthusiasm of the individual institutions.

*Patients' Contributions to Hospitals.*

Hitherto, in alluding to the crisis in the voluntary hospital system and the projected amalgamation in Bristol, we have made no allusion to the obvious fact that while the voluntary contributions of the charitable public are inadequate to maintain the increasing need for hospital accommodation, a new factor has come into existence, viz. the very large percentage of those who, when requiring hospital treatment, are well able to contribute at least a part of the cost of their maintenance. It is not right or reasonable to ask the philanthropic to pay for those who can provide for themselves, the majority of even relatively poor hospital patients would gladly contribute according to their means, and in this way hospitals already receive very considerable help in meeting their expenses.

*Paying Hospitals.*

A very urgent matter for consideration is the provision of hospital accommodation for the very large class of patients who are unable to pay the necessarily heavy charges of private nursing homes and the cost of pathological investigation, X-rays, etc., etc., in addition to medical or surgical fees, and yet are not so poor as to be proper inmates of a charitable institution. Their lot is a hard one, for on the one hand they ought not to take advantage of the hospital accommodation provided for those who are less well off, and on the other hand they often find it impossible to incur the necessary expense involved in their own homes or in private homes. We want in Bristol a paying hospital somewhat on the lines of St. Chad's in Birmingham, where the net cost of maintenance and treatment is immensely reduced by having a large number of patients, and where the fees of the medical staff are on a reduced scale. St. Chad's Hospital is maintained by the payments of the patients, and it has proved a highly successful means of filling the gap to which we direct attention. The existing hospitals cannot provide adequately for the class of patient we now refer to, seeing that they cannot even meet the requirements of the proper hospital class. A separate hospital in this city on the lines of St. Chad's would be a great boon; but although the necessity for such an institution has long been recognised, nothing hitherto has been done to provide it, and it is high time that the matter was brought to a practical issue.

